# Next-Generation Neuromodulation for Anxiety: Circuit-Based and Personalized Approaches

**DOI:** 10.1007/s11920-026-01702-3

**Published:** 2026-07-16

**Authors:** Caroline M. O’Rourke, Robert L. Selheimer, Casey H. Halpern, Desmond J. Oathes

**Affiliations:** 1 Department of Psychiatry, Perelman School of Medicine, University of Pennsylvania, Philadelphia, PA USA; 2 Department of Psychiatry, Dell Medical School, University of Texas, Austin, TX USA; 3 Department of Neurosurgery, Perelman School of Medicine, University of Pennsylvania, Philadelphia, PA USA; 4 Department of Surgery, Corporal Michael J. Crescenz Veterans Affairs Medical Center, Philadelphia, PA USA

**Keywords:** Anxiety Disorders, Neuromodulation, Deep Brain Stimulation (DBS), Transcranial Magnetic Stimulation (TMS)

## Abstract

**Purpose of Review:**

We provide an update on the current psychiatric research for DBS and TMS and what the field has learned about causal circuitry implicated in anxiety and anxiety-related disorders.

**Recent Findings:**

Recent refinement of DBS and TMS techniques have both improved their safety and efficacy as anxiety treatments and elucidated the pathological circuitry and areas causally involved in psychiatric disease.

**Summary:**

Anxiety is a debilitating hallmark of many psychiatric disorders. A significant portion of patients do not achieve sufficient benefit from psychotherapy and medication, so other therapies are being investigated. Neuromodulation, which involves targeted stimulation of neural circuitry, is FDA cleared for obsessive compulsive disorder (OCD) and depression and is an option for some patients whose anxiety have not responded to standard therapy. Over the past several years, neuromodulation protocol and technology advancements have continued to evolve the psychiatric treatment paradigm. Transcranial magnetic stimulation (TMS) and deep brain stimulation (DBS) are two of the most extensively studied neuromodulation techniques in psychiatry.

## Introduction

Anxiety is a feature of many common psychiatric diagnoses. In addition to the anxiety disorders – generalized anxiety disorder (GAD), panic disorder, social anxiety disorder, and specific phobias – the depressive disorders, obsessive-compulsive and related disorders, and trauma and stressor-related disorders all frequently endorse pathological anxiety as a prominent symptom cluster [1]. These anxiety-related disorders are among the most prevalent mental illnesses worldwide, with an estimated 25–30% of people experiencing elevated anxiety [1–7]. Significant anxiety-related disease burden necessitates investigation to find both effective and accessible treatments.

Current standards of care for the anxiety and related disorders are pharmacotherapy, psychotherapy, or a combination. For over three decades, clinical guidelines have reinforced anti-depressants, typically the selective serotonin reuptake inhibitors (SSRIs) or serotonin norepinephrine reuptake inhibitors (SNRIs), as first-line pharmacological agents given their safety and tolerability profiles [8]. Cognitive behavioral therapy (CBT), including exposure-based protocols, is considered the gold standard nonpharmacological treatment for most of these indications, whether alone or in combination with psychopharmacology. While these treatments provide significant symptom relief for many patients, many others experience only a partial or transient response. Between 50 and 67% of patients with anxiety disorders achieve partial response after the first treatment trial [9–15], and, similarly, 40–60% of patients with obsessive-compulsive disorder (OCD) attain partial therapeutic effect [16–20].

Considering these data, a significant unmet need for durable anxiety treatment remains. One class of promising interventions is neuromodulation, in which targeted stimulation (typically, electromagnetic) is delivered to a specific brain region with the goal of altering neural activity. As early as the 1940s, neuromodulation tools have been explored for their psychiatric treatment potential, including invasive methods requiring surgical implantation like deep brain stimulation (DBS) and, later, non-invasive methods like transcranial magnetic stimulation (TMS) [21, 22]. A few foundational clinical trials [23–26] solidified the treatment potential of neuromodulation for psychiatric illness, leading to U.S. Food and Drug Administration (FDA) clearance of TMS for MDD in 2008 and OCD in 2018, along with the approval of DBS for OCD in 2009 under an FDA Humanitarian Device Exemption. These promising safety and efficacy findings have also inspired exploratory off-label studies for PTSD, other OCD-like disorders, and anxiety disorders.

Although TMS and DBS have achieved FDA approval for some psychiatric indications, their mechanisms of action are not fully understood. While researchers investigate the mechanistic effects, neuromodulation studies have concurrently helped elucidate psychiatric disease pathophysiology. Growing consensus among many investigators supports the idea that specific symptoms of psychiatric disorders are driven by dysfunction in identifiable brain circuits. In this review, we focus on the two most extensively studied, focal neuromodulation tools: TMS and DBS. In these two sections, we provide a more in-depth review of each method, including current clinical use. We discuss recent, novel applications that, with further validation, could be more widely implemented given findings of improved response rates, durability, and/or safety compared to older TMS or DBS protocols. We also discuss what the field has learned about psychiatric disease circuits through these exploratory studies, with the growing focus on personalized neuromodulation given recent evidence of clinical and neural response variability between individuals. 

## Transcranial Magnetic Stimulation (TMS)

TMS is a non-invasive neuromodulation technique that involves placement of an electromagnetic coil over the scalp to target a specific region of the cortex. The TMS machine produces a magnetic field that modulates electrical activity in the targeted brain region. The most established form of TMS is repetitive TMS (rTMS), during which trains of pulses are delivered in rapid succession to the same target. Repetitive TMS targeting the dorsolateral prefrontal cortex (dlPFC) first received FDA clearance for a psychiatric indication—treatment-resistant depression—in 2008. A 58.0% response rate and 37.1% remission rate were reported by a multisite, naturalistic, observational study designed to assess TMS outcomes in clinical practice [27]. Response was defined as reaching an endpoint rating of 3 or less on the Clinician Global Impressions-Severity of Illness (CGI-S), while remission was defined as reaching an endpoint rating of 2 or 1 on the same scale. BrainsWay’s deep TMS (dTMS) targeting the dorsomedial prefrontal cortex and anterior cingulate cortex was approved for treatment-resistant obsessive-compulsive disorder (OCD) in 2018, with Carmi et al. [23] reporting response rates – defined as a ≥35% reduction on the Yale-Brown Obsessive Compulsive Scale (Y-BOCS) – of 38.1% post-treatment and 45.2% at the one-month follow up posttreatment, both significantly greater than sham treatment response rates. It is important to note that TMS has not only showed therapeutic promise in anxiety and anxiety-related disorders, but also in other psychiatric indications like skin picking [28] and substance use disorders [29, 30] and various neurological disorders, including stroke rehabilitation [31] and neurodegenerative diseases [32, 33].

The anxiolytic potential of TMS was first reported in studies of patients with MDD and co-occurring anxiety. High-frequency (i.e., excitatory) TMS over the left dorsolateral prefrontal cortex (dlPFC), the standard TMS target for depression, has been shown to relieve anxiety symptoms in patients with MDD [34–36]. Hypoactivity of the left dlPFC is considered a contributor to pathological anxiety due to deficient top-down emotional control and resultant emotional dysregulation [37–41]. Other studies have confirmed significant reductions in anxiety in response to high-frequency TMS over the left dlPFC in adults with mood and anxiety disorders. However, right-sided, low-frequency (i.e., inhibitory) TMS – in addition to bilateral stimulation [42] – is more commonly applied in the context of anxiety disorders [43] given promising research findings [39, 44, 45] and evidence of association between right dlPFC hyperactivity and excessive worrying [46].

To date, TMS is not approved for treatment of any anxiety disorder. While a handful of one-sided and bilateral dlPFC-targeted TMS studies have demonstrated significant anxiety relief, overall response rates are inconsistent, with studies citing rates from 46 to 100% [34, 35, 47–51]. Recent efforts to improve the efficacy of TMS have found distinct, circuit-based targeting of specific symptoms to be compelling. For example, Siddiqi et al. have demonstrated that anxiety experienced by patients with MDD responds to the stimulation of different TMS sites as compared to other symptoms (e.g., dysphoric) in both prospective [52] and retrospective data sets [53]. Prospective symptom-specific targeting is currently being evaluated in an ongoing clinical trial including patients with GAD [54]. In addition to a shift to more precise targeting, widespread adoption of TMS will likely benefit from practical and accessible treatment paradigms. We will summarize a few of the potentially impactful emerging trends we find most promising regarding both efficacy and accessibility. These methods will hopefully lead to a clear regulatory approval path for anxiety-targeting TMS. 

### An Interleaved TMS/fMRI-Guided Protocol: Target Optimization and Prognostic Indicator

Recognized methods of localizing selected TMS targets in current clinical practice include scalp-based measurements (most common), MRI-guided neuronavigation, and fMRI-guided targeting. Although still more commonly used in research settings, fMRI-guided TMS could replace scalp-based targeting in practice given recent evidence from our prospective study as well as retrospective studies of the superiority to scalp-based measuring and significance of target site functional connectivity in TMS response [55–58]. For example, resting-state fMRI data have shown that functional connectivity between TMS target (e.g., left dlPFC in depression) and subcortical areas of interest (e.g., subgenual anterior cingulate (sgACC) for depression) can predict clinical response [59–63] (though see [64] and [56]), supporting the potential shift in the clinic of applying fMRI to optimize target site selection. Regarding modulation of cortical-subcortical connections specific to anxiety, our 2022 study in 45 healthy participants demonstrated that fMRI-guided stimulation of the vlPFC generated changes in the amygdala fMRI BOLD signal. This work [65] shows vlPFC-amygdala communication and indirect amygdala modulation, highlighting the utility in exploring the therapeutic promise of fMRI-guided TMS in patients with anxiety.

Recently, our group has measured TMS-evoked fMRI signal response to detect individual differences in prefrontal to sgACC circuit engagement purported to be relevant to MDD [64, 66]. As we predicted, pre-intervention TMS-evoked fMRI response magnitude in the sgACC predicted depression improvement, providing support for its use as a prognostic indicator [64]. These results support the growing evidence that causal, circuit-based targeting can effectively engage and modulate deep and distributed brain regions relevant to psychopathology. 

### Mapping and Targeting Causal Circuits

Another recent focus of investigation is the treatment potential of circuit-based TMS targeting for transdiagnostic anxiety [67, 68]. As part of this work, Dr. Siddiqi’s group at Harvard mapped a normative anxiety circuit, via methods validated during previous causal circuit generation for depression [69], to determine whether TMS site connectivity to this circuit predicted change in anxiety [68]. As hypothesized, the group found that individual TMS site connectivity to a normative anxiety circuit predicted TMS-induced change in anxiety, both in patients with baseline anxiety disorders and those without (controlling for depression and variability in functional connectivity) [53, 68]. This work also showed that the circuit was specific to pathological, or trait, anxiety, versus situational anxiety (i.e., “state” anxiety, typically considered a contextually appropriate response) in two datasets that measured both metrics. Growing evidence of TMS-responsive, symptom-specific circuits support the hypothesis that mapping causal circuitry may help to identify novel, symptom-specific stimulation targets (e.g., transdiagnostic anxiety) [67]. These data and prior work seem to suggest that circuit connectivity, rather than location of target site, is the most significant parameter when considering level of response. 

### Rapid-Delivery TMS

Patient access to convenient TMS treatment also needs to be addressed. Conventional TMS protocols require weeks of daily clinical visits, limiting the number of patients who can be treated per day and impeding widespread implementation. A more recently developed protocol aiming to reduce treatment time is known as Stanford Accelerated Intelligent Neuromodulation Therapy (SAINT), an accelerated, fMRI-guided protocol, approved in 2022 and cleared for use in adolescents with MDD in 2024. This next-generation protocol uses an emerging type of rTMS called intermittent theta burst stimulation (iTBS), which delivers an equivalent dose more quickly than conventional, 10 Hz rTMS sessions. As a more efficient alternative, iTBS has been increasingly implemented given recent findings of comparable efficacy to rTMS [70], including comparably and significantly reducing anxiety symptoms in patients with anxiety and depression when applied to the left dlPFC [71, 72]. In addition, iTBS allows for a more compressed stimulation course with some accelerated protocols delivering eight to ten sessions per day (as opposed to standard once daily treatment). This has allowed for investigation of five-day TMS protocols instead of a six- to eight-week treatment course characteristic of conventional protocols [73, 74]. A small pilot study using a five-day TMS protocol recently demonstrated significant anxiety symptom reduction in Chinese patients with an anxiety disorder [75]. This preliminarily supports the efficacy of iTBS stimulation to the dorsomedial PFC (anxiosomatic circuit) for anxiety. 

### Next-Generation TMS Protocols

In addition to accelerated delivery, researchers have explored other ways to modify conventional TMS protocols. For example, Voon et al. have investigated a protocol, called cortical paired associative stimulation (cPAS), that explicitly targets interregional connectivity [76]. By mimicking spike-timing-dependent plasticity mechanisms, cPAS aims to induce plasticity changes within the targeted circuit via timed delivery of TMS pulses to two functionally connected cortical regions. Voon et al. have applied cPAS to modulate response inhibition in patients with psychiatric diseases like OCD and alcohol use disorder (AUD). They have recently shared findings that, while cPAS to healthy controls improved response inhibition, cPAS to subjects with AUD induced no improvement, indicating impaired plasticity of the targeted fronto-striatal inhibitory network in this patient population [77]. This protocol can both elucidate disease networks with impaired plasticity and may also be beneficially applied to healthy subjects and specific patient populations whose disease pathophysiology involves behavioral inhibition and compulsion. Voon’s group also discusses the use of cPAS findings as a disease biomarker, as mentioned in their recent paper on cPAS-targeted AUD. This next-generation protocol is just one of many emerging applications of TMS for psychiatric disease. 

## Deep Brain Stimulation (DBS)

### Introduction

Deep brain stimulation (DBS) is a neurosurgical treatment in which electrodes are implanted into specific deep brain regions and connected to an implantable pulse generator placed subcutaneously to deliver chronic electrical stimulation [78, 79]. Through chronic electrical stimulation, DBS modulates activity within targeted circuits, with proposed mechanisms including disruption of pathological signaling, modulation of network oscillations, and facilitation of adaptive neuroplasticity [80]. DBS is currently approved for several neurological indications, including Parkinson’s disease, essential tremor, dystonia, and epilepsy [81–84]. Given its success in modulating circuit dysfunction across these non-psychiatric conditions, DBS has increasingly been investigated as a potential therapeutic strategy for treatment-refractory psychiatric disorders, including OCD, MDD, PTSD, and other anxiety-related syndromes [85–90].

The most established psychiatric use of DBS is for OCD. DBS [85] to the anterior limb of the internal capsule [10] has built on ablative neurosurgeries targeting the cingulum and internal capsule for treatment-resistant OCD [91, 92]. DBS is considered a reversible and adjustable alternative to these ablative surgeries with similar efficacy and improved risk profile [93, 94]. After convincing pilot studies, ventral ALIC (vALIC) DBS was FDA-approved for treatment-resistant OCD in 2009 under a Humanitarian Device Exemption [95].

In parallel with this clinical OCD experience, DBS has been extensively investigated for the treatment of major depressive disorder, particularly treatment-resistant depression (TRD). Open-label trials and meta-analyses have reported the efficacy of DBS for TRD when applied to the subgenual cingulate, vALIC, and median forebrain bundle (MFB) [86]; however, outcomes of randomized controlled clinical trials have been variable, and response rate, typically defined as ≥50% reduction in the Hamilton Depression Rating Scale (HDRS) [96], is generally reported around 40–70%, with highly variable remission rates [96–99]. Of note, two industry-sponsored, double-blind, randomized trials, Reclaim [87] and BROADEN [88], both failed to show a significant difference between active and sham response rate during interim analyses. However, open-label 18-month and 24-month outcomes demonstrated more promising results with response rates near 50% [88]. Although DBS for TRD has yet to receive regulatory approval, a robust, multi-center trial (NCT06423430) is currently exploring the efficacy of bilateral stimulation of the subgenual cingulate [100].

The clinical evidence base for DBS in PTSD remains limited relative to OCD and depression. First reports of DBS for treatment-refractory PTSD describe bilateral stimulation of the basolateral nucleus of the amygdala [89] and stimulation of the medial prefrontal cortex/uncinate fasciculus [90]. Interest in applying DBS to PTSD stems not only from growing success in other anxiety-related disorders but also from neuroanatomical observations linking brain injury patterns to PTSD vulnerability. A pivotal study by Koenigs et al. [101] found that Vietnam War veterans with penetrating brain injuries involving the amygdala never developed PTSD, whereas those with lesions sparing the amygdala and ventromedial prefrontal cortex (vmPFC) had PTSD rates comparable to other combat-exposed veterans [101]. These findings align with preclinical research implicating amygdala hyperactivity and disrupted vmPFC-amygdala connectivity in PTSD pathophysiology [102–104]. Preliminary studies have since explored DBS targeting the basolateral amygdala (BLA) [105, 106] and sgACC [107], suggesting potential safety and therapeutic benefit. Notably, both targets have also been implicated in the pathophysiology of depression [108, 109] and OCD [110, 111], suggesting the idea that shared fronto-limbic circuits underlying pathological anxiety may offer translational relevance for DBS in PTSD. However, these results remain preliminary, and further investigation is necessary to determine optimal stimulation targets and long-term outcomes.

Despite promising results, DBS outcomes across anxiety-related psychiatric disorders remain highly variable in both symptom reduction and consistency of response. In OCD, response rates range between 50 and 70%, depending on the stimulation target [112]. Recent advances offer promising strategies to improve both the consistency (i.e., the response rate) and magnitude (i.e., symptom reduction) of DBS outcomes. One encouraging approach, informed by network theory, involves circuit-based targeting with a focus on conserved symptom-specific brain pathways associated with optimal clinical outcomes. Other data-driven approaches, such as neuroimaging and electrophysiology, enhance efficacy by guiding preoperative target and parameter selection, predicting response, and enabling postoperative biomarker tracking. The most innovative and potentially impactful strategies include the responsive DBS (rDBS) systems – these adapt stimulation parameters in real time, with the potential to both improve efficacy and reduce side effects.

### Circuit-Based Targeting & Symptom-Specific Pathways

Over the past two decades, the understanding of how DBS exerts therapeutic effects in psychiatric illness has shifted from targeting isolated anatomical regions to modulating broader brain networks. This shift is supported by findings that DBS to a given region can improve a particular symptom (e.g., mood) in both OCD and depression, suggesting convergence on common functional circuits [24, 113]. Network-based models emphasize the importance of symptom-specific pathways rather than single anatomical nodes. For example, in OCD, target sites such as the ventral capsule/ventral striatum (VC/VS) and nucleus accumbens (NAc) are now understood to engage shared white matter tracts within the cortico-striato-thalamo-cortical (CSTC) circuit—long implicated in OCD pathophysiology [113, 114].

This framework has given rise to novel analytic approaches, including “sweet-spot” mapping and tractography-guided targeting. In sweet spot mapping, researchers retrospectively examine volumes of tissue activated (VTAs) in patients with varying clinical outcomes to identify stimulation sites most associated with symptom improvement. These sweet spots are then mapped onto a normative structural connectome (i.e., a brain template containing known white matter tracts) to determine which white matter tracts and broader brain networks are being modulated [115–118]. Tractography-based methods use preoperative diffusion-weighted MRI to model individual patients’ white matter connections and determine which fiber pathways are most associated with therapeutic response [119, 120].

Several connectomic studies [113, 121, 122] using this fiber-filtering approach to analyze clinical response in OCD cohorts have converged on a specific fiber bundle connecting the medial and lateral prefrontal cortex to subcortical regions such as the subthalamic nucleus (STN) and the mediodorsal thalamus. Notably, a “hyperdirect pathway” connecting the dorsal anterior cingulate cortex (dACC) with the STN via the ALIC [122] has emerged as a particularly promising target – consistent with both previous data [113, 123–125] and the rationale for existing neuromodulation techniques like TMS that focus on dACC and medial prefrontal regions [23].

This network-informed strategy has also been applied to depression. Probabilistic tractography has been used to identify optimal sgACC DBS targets by reconstructing white matter pathways most predictive of response, including the cingulum bundle and forceps minor [126–128]. Transitioning from anatomical landmark-based targeting to tractography-guided approaches has led to improved outcomes in treatment-resistant depression and further supports a shift toward individualized, circuit-based DBS protocols [129].

### Data-Driven Approaches to DBS for Anxiety-Related Psychiatric Disorders: Neuroimaging and Electrophysiology

To improve patient selection, target precision, and stimulation parameters, recent studies have increasingly incorporated data-driven methods, particularly neuroimaging and electrophysiology. These tools aim to identify biomarkers that predict treatment response and guide more personalized DBS strategies.

Neuroimaging has also been used to identify potential treatment responders. For instance, sgACC DBS responders with depression have shown higher baseline glucose metabolism [130] and increased regional volume [131] in the sgACC compared to non-responders. Similarly, in OCD, greater baseline volume of the nucleus accumbens – a common DBS target – has been associated with better clinical response [132]. These findings suggest that neuroimaging biomarkers may help predict which patients are most likely to benefit from DBS.

Electrophysiological recordings, particularly electroencephalography (EEG) and local field potentials (LFPs), have been used with connectomic modeling to further characterize brain responses to DBS. In sgACC DBS for depression, studies have identified scalp EEG-derived evoked potentials that vary based on which white matter tracts are activated—such as the forceps minor versus cingulum bundle—supporting the idea of tract-specific brain responses [128]. More recently, chronic LFP recordings from implanted DBS leads have revealed potential biomarkers of treatment response. For example, a unique LFP signature in the sgACC was found to distinguish early behavioral changes from sustained recovery, correlating with facial expression and neuroimaging metrics [108]. In a separate study, elevated beta power in the right sgACC was linked to clinical improvement [133]. 

Efforts to apply electrophysiology to OCD are ongoing. Preliminary research has explored the role of cortical theta power as a predictor of clinical state and its modulation by ventral ALIC DBS voltage, although findings remain inconclusive [134–136]. Together, these neuroimaging and electrophysiological tools are helping refine DBS approaches by offering individualized insights into optimal targets and response prediction—an essential step toward making DBS more effective and scalable in psychiatric practice.

### Responsive DBS: A Move Towards a Closed-Loop System

Recent ongoing clinical trials of depression and OCD DBS have introduced the application of intracranial EEG (iEEG), an electrophysiology modality typically used in the epilepsy monitoring unit for seizure localization. This technique involves temporarily implanting electrodes in several brain areas to map circuits for neural networks implicated in the pathophysiology of the indicated psychiatric disease. Multiple groups have shown the utility of using iEEG recordings, stimulus response mapping, and fiber pathway mapping to develop personalized, closed-loop DBS for depression [137–139]. Another trial (NCT05623306) with a similar objective and protocol uses iEEG to map neural circuits for OCD networks.

While iEEG, or stereoelectroencephalography (SEEG), paired with neurophysiology, neuroimaging, and computational models of stimulation has demonstrated the ability to guide patient-specific targeting and parameter selection (i.e., more personalized DBS), it may also improve our understanding of underlying disease pathophysiology and guide the development of a fully closed-loop DBS device. This device, a responsive DBS system, has gained recent appeal given its potential to make real-time parameter adjustments based on electrophysiological biomarkers that reflect the patient’s symptom fluctuations. A closed-loop DBS system that automatically adjusts to biomarker activity in the amygdala has been implemented in an MDD patient with significant symptom improvement [138]. Similarly, a case report shared promising first-in-human findings of a responsive DBS strategy for OCD [140] and a pilot study of responsive stimulation in PTSD demonstrated improvement in symptoms [106].

## Conclusions

Anxiety remains one of the most pervasive and burdensome symptom domains across psychiatric disorders, and current first-line treatments – while effective for many – leave a substantial portion of patients without meaningful or lasting relief. Neuromodulation techniques such as TMS and DBS offer promising alternatives, especially for individuals with treatment-resistant illness. As reviewed here, both modalities have evolved beyond early exploratory use, with recent innovations improving precision, durability, and mechanistic understanding.

TMS, a non-invasive and increasingly accessible intervention, has demonstrated anxiolytic effects in multiple psychiatric populations. Emerging paradigms—including fMRI-guided targeting, causal circuit mapping, and accelerated stimulation protocols—suggest pathways toward regulatory approval and broader clinical use for anxiety-specific indications.

Meanwhile, DBS offers a precise, circuit-based option for severe, refractory cases, particularly in OCD and depression. Recent advances—particularly in network-based targeting, neuroimaging and electrophysiological biomarkers, and responsive DBS systems—have helped shift the field toward more consistent, individualized approaches. However, as emphasized by Davis and colleagues [141], substantial barriers remain to broad clinical adoption. These include limited insurance coverage, geographic disparities in specialized care, stigma, and the need for sustained interdisciplinary follow-up. Bridging the gap between scientific innovation and real-world accessibility will require coordinated efforts in clinical research, implementation science, and health policy. Ultimately, realizing the full therapeutic potential of DBS will depend on both identifying the right circuits and building the infrastructure to reach the right patients.

Importantly, both TMS and DBS contribute not only therapeutic tools but also causal insights into the neural circuits underlying pathological anxiety. The field is now moving toward a paradigm that values individualized, symptom-specific neuromodulation strategies grounded in mechanistic precision. Achieving broader impact, however, will require attention to practical barriers—including access, reimbursement, and care infrastructure—alongside continued research. As the science matures, integration of these modalities into clinical psychiatry may transform how we understand and treat anxiety across diagnostic boundaries.

Lastly, while both TMS and DBS have evolved beyond early exploratory use, other neuromodulation methods, such as magnetic seizure therapy (MST) [22] and transcranial focused ultrasound (tFUS), have just begun to emerge in the field. Lisanby and colleagues published data last year from their robust, randomized clinical trial, demonstrating MST’s comparable efficacy and superior safety to electroconvulsive therapy (ECT)—an established depression therapy—for depression [142]. Additionally, Barksdale et al. demonstrate the capability of tFUS, which noninvasively delivers low-intensity sound waves with precise targeting capability to deep brain regions, to produce significant primary and secondary outcome differences among active vs. sham patients with (transdiagnostic) mood, anxiety, and trauma-related disorders [143]. Overall, it is important to remain aware and optimistic regarding neuromodulation as a treatment for psychiatric disease.

## Data Availability

No datasets were generated or analysed during the current study.

## Key References

- Carmi L, Tendler A, Bystritsky A, Hollander E, Blumberger DM, Daskalakis J, et al. Efficacy and Safety of Deep Transcranial Magnetic Stimulation for Obsessive-Compulsive Disorder: A Prospective Multicenter Randomized Double-Blind Placebo-Controlled Trial. Am J Psychiatry. 2019;176(11):931–8.
  - This pivotal, multicenter study established dTMS as the first FDA-cleared neuromodulation method for OCD and highlighted its potential for patients who were refractory to pharmacological interventions.
- Cole EJ, Phillips AL, Bentzley BS, Stimpson KH, Nejad R, Barmak F, et al. Stanford Neuromodulation Therapy (SNT): A Double-Blind Randomized Controlled Trial. Am J Psychiatry. 2022;179(2):132–41.
  - The Stanford Neuromodulation Therapy (SNT) clinical trial validated the efficacy of an accelerated, high-dose iTBS protocol for treatment-resistant depression that received FDA clearance in 2022. This protocol has been increasingly prescribed over conventional TMS protocols given its greater efficiency and convenience for patients.
- Siddiqi SH, Taylor SF, Cooke D, Pascual-Leone A, George MS, Fox MD. Distinct Symptom-Specific Treatment Targets for Circuit-Based Neuromodulation. The American journal of psychiatry. 2020;177(5):435–46.
  - The retrospective analyses of this study provided evidence that in depression patients distinct symptom or symptom clusters respond to different TMS targets with different circuitry. These data incited an ongoing prospective trial and solidified the potential of personalized stimulation targets.
- Fox MD, Buckner RL, White MP, Greicius MD, Pascual-Leone A. Efficacy of Transcranial Magnetic Stimulation Targets for Depression Is Related to Intrinsic Functional Connectivity with the Subgenual Cingulate. Biological Psychiatry. 2012;72(7):595–603.
  - This work confirms a relation between clinical antidepressant efficacy with the degree of TMS site anticorrelation to the subgenual cingulate. It helps define the mechanism of TMS for depression and the potential value in selecting TMS targets based on their underlying circuitry or functional connectivity.
- Siddiqi SH, Schaper F, Horn A, Hsu J, Padmanabhan JL, Brodtmann A, et al. Brain stimulation and brain lesions converge on common causal circuits in neuropsychiatric disease. Nat Hum Behav. 2021;5(12):1707–16.
  - Robust, retrospective analyses of brain lesions, TMS sites, and DBS sites demonstrate their convergence on common brain circuitry based on association of these lesions or sites with depression severity. The protocol outlined in this paper was also used to determine a similar convergence of lesion/site circuitry for transdiagnostic anxiety, indicating that target selection for these psychiatric diseases may be improved by using this method to define disease-specific brain circuits.
- Meyer GM, Hollunder B, Li N, Butenko K, Dembek TA, Hart L, et al. Deep Brain Stimulation for Obsessive-Compulsive Disorder: Optimal Stimulation Sites. Biol Psychiatry. 2024;96(2):101–13.
  - Outlined in this paper, sweet-spot mapping retrospectively finds DBS stimulation sites that are positively or negatively correlated with OCD clinical improvement. The sites are then mapped to a connectome to determine broader connectivity, one of the more recent circuit-based methods to optimizing DBS target selection.
- Tyagi H, Apergis-Schoute AM, Akram H, Foltynie T, Limousin P, Drummond LM, et al. A Randomized Trial Directly Comparing Ventral Capsule and Anteromedial Subthalamic Nucleus Stimulation in Obsessive-Compulsive Disorder: Clinical and Imaging Evidence for Dissociable Effects. Biol Psychiatry. 2019;85(9):726–34.
  - This DBS trial is the first to compare two different target sites (VC/VS and amSTN) within the same patients. Results show significant, comparable OCD symptom reduction between the sites while also indicating site-specific effects on mood and cognitive flexibility. Probabilistic tractography with modelled volumes of tissue activation (VTAs) around optimally active DBS electrode contacts as seeds provides evidence that the different DBS targets engage separate circuits.
- Provenza NR, Reddy S, Allam AK, Rajesh SV, Diab N, Reyes G, et al. Disruption of neural periodicity predicts clinical response after deep brain stimulation for obsessive-compulsive disorder. Nat Med. 2024;30(10):3004–14.
  - Analyzing intracranial recordings of OCD patients, this research demonstrates distinct patterns in cortical theta power as a predictor of clinical state. The results indicate the potential use of neural data as both predictors of clinical state and markers of DBS response.
- Nho Y-H, Rolle CE, Topalovic U, Shivacharan RS, Cunningham TN, Hiller S, et al. Responsive deep brain stimulation guided by ventral striatal electrophysiology of obsession durably ameliorates compulsion. Neuron (Cambridge, Mass). 2024;112(1):73.
  - This case report of first-in-human findings of a responsive DBS strategy in an OCD patient has helped to justify the treatment potential of a closed-loop DBS system for OCD.
- Barksdale BR, Enten L, DeMarco A, Kline R, Doss MK, Nemeroff CB, et al. Low-intensity transcranial focused ultrasound amygdala neuromodulation: a double-blind sham-controlled target engagement study and unblinded single-arm clinical trial. Molecular Psychiatry. 2025.
  - Findings from a single-arm clinical trial in patients with mood, anxiety, or trauma-related disorders indicate the potential efficacy and feasibility of directly targeting subcortical regions (e.g., amygdala) with low-intensity transcranial focused ultrasound (tFUS), a novel non-invasive neuromodulation technique.
